# Ergothioneine-Derived Carbon Dot Nanozymes Alleviate the Oxidative Stress Microenvironment to Delay Intervertebral Disc Degeneration

**DOI:** 10.3390/antiox15091082

**Published:** 2026-08-28

**Authors:** Ziyang Zhang, Kehan Wang, Hao Chen, Yu Shi, Huihui Wang

**Affiliations:** 1 The First School of Clinical Medicine, Faculty of Medicine, Yangzhou University, Yangzhou 225009, China; zzy11912024@163.com (Z.Z.); khanremb0511@163.com (K.W.); hchen2020@yzu.edu.cn (H.C.); 2 School of Traditional Chinese Medicine, Faculty of Medicine, Yangzhou University, Yangzhou 225009, China; 3 Key Laboratory of the Jiangsu Higher Education Institutions for Nucleic Acid & Cell Fate Regulation, Yangzhou University, Yangzhou 225009, China

**Keywords:** ergothioneine-derived carbon dots, nanozyme, nucleus pulposus cells, oxidative stress, intervertebral disc degeneration

## Abstract

Nucleus pulposus cells (NPCs) age mainly because reactive oxygen species (ROS) build up too much. Excess ROS is associated with intervertebral disc degeneration (IVDD). In this study, we prepared ergothioneine-derived carbon dots (EGT-Fe-CDs) as an antioxidant nanozyme. EGT-Fe-CDs showed good biocompatibility, superoxide dismutase-like and catalase-like activities, and high total antioxidant capacity. In H_2_O_2_-treated NPCs, EGT-Fe-CDs reduced cell damage. Local EGT-Fe-CDs treatment also reduced degeneration in the animal model, based on imaging, disc height index (DHI), and Pfirrmann grade. EGT-Fe-CDs also reduced mitochondrial damage associated with excess ROS. They slow down NPC ageing and help control IVDD. Based on these characteristics, the nanozyme could be a useful option for clinical therapy in the future.

## 1. Introduction

Intervertebral disc degeneration (IVDD) is a huge health problem worldwide. It severely lowers the quality of life for patients and also costs healthcare systems a lot of money [1]. Doctors see this condition very often in the clinic. But we still do not fully understand how it starts. Current treatments mostly just manage the pain. They cannot actually fix or reverse the disease [2,3,4,5]. We need to find new targets for therapy. Hence, learning more about the molecular details of IVDD is very important for both science and medicine. Recently, many reports point to reactive oxygen species (ROS). ROS are important in the signalling networks of intervertebral disc cells [6,7,8]. These are very active molecules that contain oxygen. Examples include hydrogen peroxide, singlet oxygen, superoxide anions, and hydroxyl radicals. They help control normal body functions and disease states. This involves processes like extracellular matrix metabolism, inflammation, apoptosis, autophagy, and ageing [9,10]. Normally, cells keep ROS in a safe balance. They generate ROS and clean it up at the same rate. Things change during bad conditions, like too much mechanical loading, inflammation, or poor nutrient supply. When this happens, disc cells make too much ROS. The cells cannot clean it all up. This buildup causes oxidative stress [11]. Oxidative stress causes a lot of damage. It turns on matrix-degrading enzymes and stops the making of new matrix. It also pushes cells to release inflammatory factors. More cells become senescent, and fewer stay healthy. All of this ruins the local environment of the disc and speeds up the degeneration process [12,13,14]. Because of this chain of events, blocking oxidative stress is seen as a good way to slow down or reverse IVDD.

Natural antioxidant enzymes exist in living things. Two common ones are superoxide dismutase (SOD) and catalase (CAT). They clean up ROS well. This protects cells from oxidative damage and slows down tissue ageing and degeneration. But using natural enzymes in real treatments is hard. They lose their function easily when the temperature or pH changes [15]. They also do not last very long in the body. This means they cannot work for a long time at the exact site of the disease. Making them pure is another big problem. The steps are complex and cost a lot of money. Because of these limits, researchers are now trying to make artificial enzymes. The goal is to copy natural enzymes but make them cheaper and more stable. Nanozymes are nanomaterials that act like regular enzymes [16,17]. Out of all the choices, carbon dot nanozymes are growing very fast right now. They are tiny, usually under 10 nm. This very small size is paired with multiple enzyme-like reactions. This mix gives them special benefits for nanomedicine [18,19,20]. Take selenium-doped carbon quantum dots (Se-CQDs) as an example. Cells take them in easily. Once inside, they drop ROS levels a lot. This fights oxidative stress and slows down ageing [21]. But not all carbon dot nanozymes are perfect. Fullerenes, for instance, do not dissolve well in liquids. They also have low biocompatibility, making them hard to use in biology. We clearly need better options. Making new carbon-dot nanozymes with high antioxidant power, good biocompatibility, and multiple enzyme activities is very important for future medical use.

L-Ergothioneine (EGT) is a natural amino acid derivative that contains sulfur. People first found it in ergot fungi in 1909. The human body cannot make EGT on its own. We have to get it from our food, unlike N-acetylcysteine [22]. When EGT enters the body, a special transporter named OCTN1 moves it. It quickly gathers in organs that easily suffer from oxidative damage, like the liver, kidneys, brain, and bone marrow. This shows that EGT targets very specific tissues naturally [23,24,25]. Right now, using EGT to build nanozymes is a very new idea. EGT is a great antioxidant. It also has a special molecular structure with a thioimidazole ring. Because of these features, we used a hydrothermal method in this study. We created a new carbon-dot nanozyme called ergothioneine-derived carbon dots (EGT-Fe-CDs). We designed this research based on how oxidative stress causes IVDD. We then tested EGT-Fe-CDs in cell and animal models of disc degeneration to assess their ROS-scavenging and antioxidant effects.

## 2. Materials and Methods

### 2.1. Materials

Ergothioneine (EGT), citric acid, ferric chloride hexahydrate (FeCl_3_·6H_2_O), and Safranin O-Fast green were purchased from Sigma-Aldrich (Burlington, MA, USA). Ethylenediamine (EDA) and the superoxide dismutase (SOD) assay kit were obtained from Sinopharm (Shanghai, China). Beyotime (Shanghai, China) supplied the total antioxidant capacity assay kit (ABTS rapid method), CCK-8 cell counting kit, Calcein-AM/PI cell viability staining kit, MitoTracker Red CMXRos probe, dihydroethidine (DHE), SA-β-Gal staining kit, DAPI dihydrochloride, 4% paraformaldehyde, H&E staining kit, and other routine cell and histology reagents. For the antibodies, the anti-aggrecan antibody was obtained from ABclonal (Wuhan, China). Proteintech (Wuhan, China) provided the anti-MMP13 and anti-TNF-α antibodies. DMEM/F12 medium and foetal bovine serum (FBS) were bought from Gibco (Waltham, MA, USA). All other basic chemicals were of analytical grade.

### 2.2. Instruments

Nanozyme microstructure was examined by transmission electron microscopy (TEM, Tecnai 12, Philips, Amsterdam, Netherlands). Hydrated particle size and surface potential were measured with a ZEN3690 nanoparticle size and zeta potential analyser (Malvern Panalytical, Malvern, UK). Crystal structure was examined using a D8 Advance X-ray diffractometer (Bruker, Billerica, MA, USA). Surface functional groups were analysed with a Cary 610/670 micro-IR spectrometer (Varian, Palo Alto, CA, USA), and surface elemental composition and chemical states were measured with an X-ray photoelectron spectrometer (ESCALAB 250Xi, Thermo Scientific, Waltham, MA, USA). To measure how well the material clears free radicals, we ran tests on a Bruker electron paramagnetic resonance (EPR) spectrometer. A Carl Zeiss fluorescence microscope (Carl Zeiss, Jena, Germany) captured images of the stained cells and tissue sections. Finally, we checked the live animals using MRI and X-ray imaging equipment (GE Medical Systems, LLC Waukesha, WI, USA).

### 2.3. Preparation of EGT-Fe-CDs

We made EGT-Fe-CDs using a basic hydrothermal method. First, we mixed citric acid (960.6 mg, 0.005 mmol), ethylenediamine (300.5 mg, 0.005 mmol), FeCl_3_·6H_2_O (270.3 mg, 0.001 mmol), and ergothioneine (229.3 mg, 0.001 mmol) into ultrapure water. We stirred this at room temperature for 30 min. Once everything was completely dissolved, the liquid went into a PTFE-lined reactor. We heated it at 180 °C for 10 h. After the heating finished, the mixture cooled down naturally to room temperature. We then used a 0.22 μm microporous filter membrane to strain out any solid waste. Next, the filtered liquid was put inside a dialysis bag (molecular weight cut-off of 500–1000 Da) to purify it. To finish, we freeze-dried the clean product. This gave us the dry EGT-Fe-CDs powder. It stayed in a desiccator until we needed it for tests.

### 2.4. Characterisation of EGT-Fe-CDs

We mixed a small amount of EGT-Fe-CDs powder into ultrapure water. The suspension was sonicated and placed on a copper mesh for TEM imaging and particle-size analysis. Dynamic light scattering (DLS) was used to measure the hydrodynamic diameter, and a zeta potential analyser was used to measure surface potential. Crystal structure was analysed by X-ray diffraction (D8 Advance, Bruker AXS, Cu Kα radiation, scanning range 10–80°). Surface functional groups in EGT and EGT-Fe-CDs were examined by Fourier transform infrared spectroscopy (FT-IR, Cary 610/670, Varian). Lastly, X-ray photoelectron spectroscopy (XPS, ESCALAB 250Xi, Thermo Scientific) showed the elemental composition and chemical states on the surface. We finished the process by doing peak fitting on the high-resolution C1s, O1s, and Fe2p spectra.

### 2.5. Enzyme-like Activities and Total Antioxidant Capacity of EGT-Fe-CDs

A total superoxide dismutase assay kit (NBT method) helped us measure the SOD-like activity. In this test, xanthine and xanthine oxidase mix to make the superoxide anion (O_2_•^−^). This anion turns nitroblue tetrazolium into a blue formazan product, which shows a strong absorption at 560 nm. EGT-Fe-CDs scavenged O_2_•^−^ and reduced blue formazan formation; a higher inhibition rate indicated stronger SOD-like activity. CAT-like activity was measured with a dissolved oxygen metre. EGT-Fe-CDs and H_2_O_2_ (0.8 M) were mixed in an acetic acid–sodium acetate buffer (pH 7.0) at 37 °C, and dissolved oxygen was recorded over time to assess H_2_O_2_ decomposition into O_2_. Total antioxidant capacity (T-AOC) was measured by the rapid ABTS method. In this assay, ABTS is oxidised to green ABTS•^+^, which has an absorption peak at 734 nm. Antioxidants block this green product from showing up. We looked at the absorbance changes to calculate the total antioxidant capacity. Finally, an EPR spectrometer was used. This extra step proved that EGT-Fe-CDs really do clear away O_2_•^−^ and hydroxyl radicals (•OH).

### 2.6. Cell Viability Assay

We put NPCs into a 96-well plate. Each well held 8000 cells. The cells grew for 24 h. Then, we tested two different treatments on them for another 24 h. Some cells got EGT-Fe-CDs (0, 1.25, 2.5, 5, 10, or 20 μg/mL), while others received H_2_O_2_ (0, 25, 50, 100, 200, or 400 μM). We also needed to know if EGT-Fe-CDs could block H_2_O_2_-induced oxidative stress damage. For this part, we gave the cells different EGT-Fe-CDs concentrations along with 100 μM H_2_O_2_ for 24 h. After finishing the treatments, we put the CCK-8 working solution into every well. The plate sat in an incubator at 37 °C. Finally, we read the absorbance at 450 nm. This number let us calculate the cell survival rate.

### 2.7. Live and Dead Cell Staining

We used a Calcein-AM/PI cell viability staining kit to check for cell death. First, the NPCs received their specific group treatments. We then washed them three times with PBS. After that, we added the calcein-AM/PI solution. The cells stayed in the dark at 37 °C for 30 min. We used a fluorescence microscope to look at the samples and take photos. In these pictures, dead cells gave off red fluorescence (PI). Live cells showed green fluorescence (calcein-AM). Finally, ImageJ 1.53t software was used to do a semiquantitative analysis on the images.

### 2.8. Assessment of Intracellular ROS Levels

We measured intracellular superoxide anion and ROS levels. A dihydroethidine (DHE) fluorescent probe was used for this step. First, the NPCs received their group treatments. We then washed them three times with PBS. Next, we added the DHE working solution (20 μM). The cells sat in the dark at 37 °C for 20 min. After that, we stained the cells with DAPI for 5 min. We washed the cells one last time. A fluorescence microscope captured the red fluorescence images. Finally, we calculated the mean fluorescence intensity (MFI) for every group.

### 2.9. Mitochondrial Membrane Potential Assay

We checked the mitochondrial membrane potential using MitoTracker Red CMXRos staining. The NPCs first received their specific treatments. Then, we washed them with PBS and added the CMXRos working solution (100 nM). The cells stayed in the dark at 37 °C for 20 min. After this step, we fixed the cells with 4% paraformaldehyde. We also stained them with DAPI to clearly visualise the nuclei. Finally, a fluorescence microscope showed us the red fluorescence intensity. This told us how well the mitochondria were working.

### 2.10. Detection of Cell Ageing

We used a senescence-associated β-galactosidase (SA-β-Gal) staining kit to check cellular senescence. First, the NPCs went through their assigned treatments. We then washed them with PBS. A fixative was applied at room temperature for 15 min. After washing the cells one more time, we added the SA-β-Gal staining solution. The samples were kept overnight in an incubator at 37 °C. The next day, a light microscope was used to observe the blue-stained cells. Finally, we calculated the percentage of positive cells based on these observations.

### 2.11. Cellular Immunofluorescence Staining

First, the NPCs finished their treatments. We fixed them in 4% paraformaldehyde for 15 min. Then, we used 0.3% Triton X-100 at room temperature to permeabilise the cells for 15 min. We washed them with PBS. Bovine serum albumin blocked the samples for 2 h. Next, we added the primary antibodies separately. We used rabbit anti-aggrecan, rabbit anti-MMP13, and mouse anti-TNF-α. The samples stayed at 4 °C overnight. The next day, we removed the primary antibodies. The samples were washed with PBS again. We then added the correct fluorescent secondary antibodies (goat anti-rabbit or goat anti-mouse IgG). These sat in the dark at 37 °C for 1 h. Finally, DAPI stained the nuclei for 5 min. We put an anti-fade mountant on the slides. A fluorescence microscope was used to view the samples and take pictures.

### 2.12. Establishment and Grouping of Rat Models

We used four-week-old male SD rats for this test. They came from the Center for Comparative Medicine at Yangzhou University. The Ethics Committee of Yangzhou University approved the work (Approval No. 2024-02101). We randomly split the 20 rats into four groups (n = 5 for each). These included a normal control group, a degeneration control group (vehicle group), a low-dose EGT-Fe-CDs treatment group, and a high-dose EGT-Fe-CDs treatment group. To put the rats to sleep, we gave them an intraperitoneal injection of sodium pentobarbital (5 mg/100 g body weight). We cleaned the skin on their tails. Next, we found and marked the intervertebral disc between the 8th and 9th caudal vertebrae (Co8-9). We made the needle-puncture degeneration model using a 21G needle. We pushed the needle straight down into the disc, turned it 360°, and held it there for 30 s. Right after taking it out, we injected 10 μL of the correct liquid into the area. The normal control group got nothing. The degeneration control group got PBS. The two treatment groups received different concentrations of the EGT-Fe-CDs solutions. The rats got standard care after the surgery. Four weeks went by. We then ran X-ray and MRI scans on them. Finally, we put the rats to sleep permanently under deep anaesthesia. We collected the Co8-9 intervertebral disc specimens to do histological and immunofluorescence analysis later.

### 2.13. Imaging Evaluation

We took X-ray pictures of the rats. First, the animals lay face down. Their tails stretched out naturally. We used X-ray imaging equipment to get lateral views. Image analysis software then helped us check the sizes. We measured the intervertebral disc height. We also recorded the heights of the adjacent upper and lower vertebral bodies. Using these numbers, we calculated the disc height index (DHI) and DHI percentage (DHI%). We followed standard methods from the literature. For the MRI tests, a 3.0T magnetic resonance imaging system was used. We ran coronal T2-weighted imaging (T2WI) scans. The machine parameters matched the exact criteria for evaluating IVDD. Finally, we scored the degree of disc degeneration. The Pfirrmann grading system (grades I–V) was applied for this step.

### 2.14. Histological Evaluation

We put the Co8-9 intervertebral disc specimens into 10% neutral formalin to fix them. Next, a 10% EDTA decalcification solution was used to thoroughly remove the calcium. We then dehydrated the tissue using a gradient of ethanol. After embedding the samples in paraffin, we cut them into consecutive coronal sections. Each slice was 5 μm thick. For staining, we used haematoxylin and eosin (H&E) on some sections. Others received Safranin O–Fast Green. Finally, we looked at the slides under a light microscope to check several details. We observed the overall structure of the intervertebral disc. We also checked the clarity of the boundary between the nucleus pulposus and the annulus fibrosus. In addition, we noted the orderliness of the annulus fibrosus arrangement. Lastly, we recorded the number and morphology of nucleus pulposus cells, along with the degree of extracellular matrix aggregation.

### 2.15. Tissue Immunofluorescence Staining

We got the paraffin sections of the intervertebral discs ready. First, they were deparaffinised in water normally. Then, we did antigen retrieval and blocking. Next, we applied the primary antibodies. These included DHE to detect tissue ROS levels, rabbit anti-MMP13, mouse anti-TNF-α, and rabbit anti-aggrecan. The tissue samples stayed at 4 °C overnight. We washed the samples the next day. After that, we put on the right fluorescent secondary antibodies. The slides sat at 37 °C for 1 h. The nuclei were stained with DAPI. We then covered the slides using an anti-fluorescence quencher. A fluorescence microscope let us check the intensity of the red or green fluorescence for every group. Finally, we ran a semiquantitative analysis using ImageJ software. This step proved the in vivo antioxidant, anti-inflammatory, and matrix-protective effects of the EGT-Fe-CDs.

### 2.16. Statistical Analysis

We ran every experiment independently at least three times. All data are shown as the mean ± standard deviation (mean ± SD). We used GraphPad Prism 9.0 software to do the statistical analysis and make the graphs. When comparing just two groups, Student’s *t* test was applied. For more than two groups, we chose a one-way ANOVA. A *p* value < 0.05 meant the result had statistical significance. The specific levels are marked as * *p* < 0.05, ** *p* < 0.01, *** *p* < 0.001, and **** *p* < 0.0001. Finally, the letters “ns” mean there is no statistically significant difference.

## 3. Results

### 3.1. Synthesis and Characterisation of EGT-Fe-CDs

**Figure 1:**
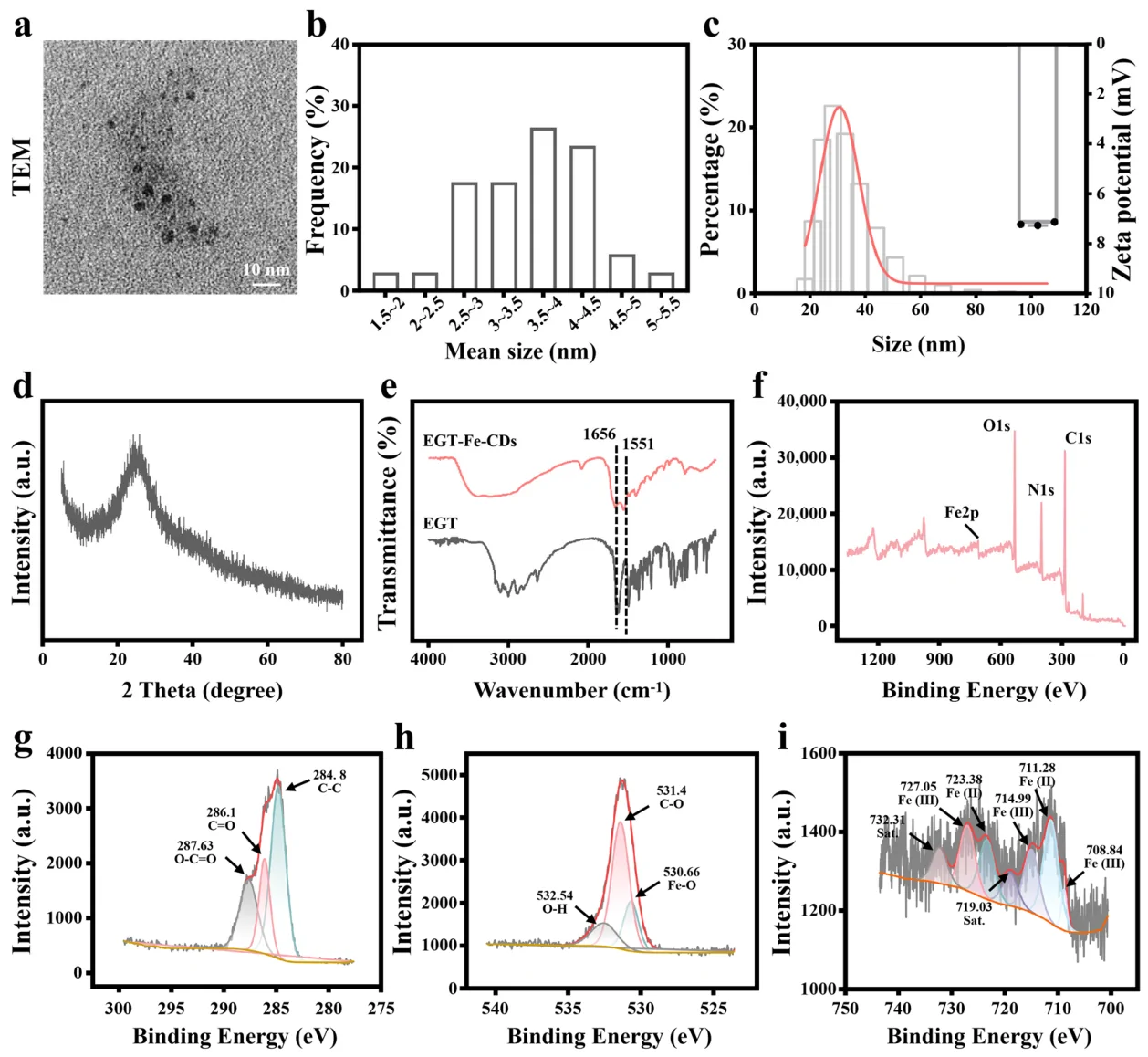
Characterisation of the EGT-Fe-CDs. (**a**) TEM image of the EGT-Fe-CDs and (**b**) size distribution (scale bar = 10 nm). (**c**) Hydrated particle size (left) and zeta potential (right). The red lines represent the fitting curves. (**d**) XRD pattern. (**e**) FT-IR spectra of EGT and EGT-Fe-CDs. (**f**) XPS survey spectrum of EGT-Fe-CDs. (**g**–**i**) High-resolution C 1s, O 1s, and Fe 2p XPS spectra, respectively.

We synthesised EGT-Fe-CDs using a hydrothermal method [26,27]. The transmission electron microscopy (TEM) images show that the particles are spread out evenly. They are almost round. Their size is about 4 nm (Figure 1a,b). Dynamic light scattering (DLS) gave an average hydrodynamic diameter of 27.65 nm (Figure 1c, left). The zeta potential was 7.59 ± 0.88 mV (Figure 1c, right). This means they mix well in water. No clear diffraction peaks were found in the X-ray diffraction (XRD) pattern. This points to the low crystallinity of the EGT-Fe-CDs (Figure 1d). We used Fourier transform infrared (FT-IR) spectroscopy on both the EGT-Fe-CDs and EGT. This step helped us find the functional groups on the surface. The absorption band around 1656 cm^−1^ in Figure 1e matches the amide I band (C=O stretching vibration), indicating that carbonyl groups exist on the EGT-Fe-CDs surface. Another band is near 1551 cm^−1^. It relates to the amide II band (N-H bending vibration + C-N stretching vibration). Because of this, it was known the EGT-Fe-CDs keep some of EGT’s original structure. We also checked the chemical makeup with X-ray photoelectron spectroscopy (XPS). The results confirm that EGT-Fe-CDs mostly contain C, N, O, and Fe (Figure 1f). We looked closely at the high-resolution C1s spectrum through peak fitting. It splits into three parts. The main peak at 284.8 eV comes from C–C/C=C bonds, which means the material has an sp^2^-hybridised carbon backbone. The 286.1 eV peak matches C=O bonds. The 287.63 eV peak is from O–C=O bonds. These findings show a lot of oxygen-containing functional groups on the EGT-Fe-CDs surface. The carbon framework is also somewhat oxidised (Figure 1g). The high-resolution O1s spectrum showed three different oxygen types. The 530.66 eV peak is linked to the Fe–O bond. This proves that iron is there as an oxide. The main peak at 531.4 eV belongs to the C–O bond. Finally, the 532.54 eV peak comes from the O–H bond. Fe–O bonds means the iron chemically connects to oxygen. It is spread through the material as iron oxide nanoparticles or iron–oxygen coordination structures (Figure 1h). The high-resolution Fe 2p spectrum gave multiple splitting peaks of Fe 2p_3_/_2_ and Fe 2p_1_/_2_. This matches the normal pattern for iron oxides. Hence, the iron in EGT-Fe-CDs is in an oxide form, not just plain iron elements. Putting all this together, these tests confirm that we successfully synthesised the EGT-Fe-CDs.

### 3.2. Antioxidant Capacity and Enzyme-like Activities of EGT-Fe-CDs

**Figure 2:**
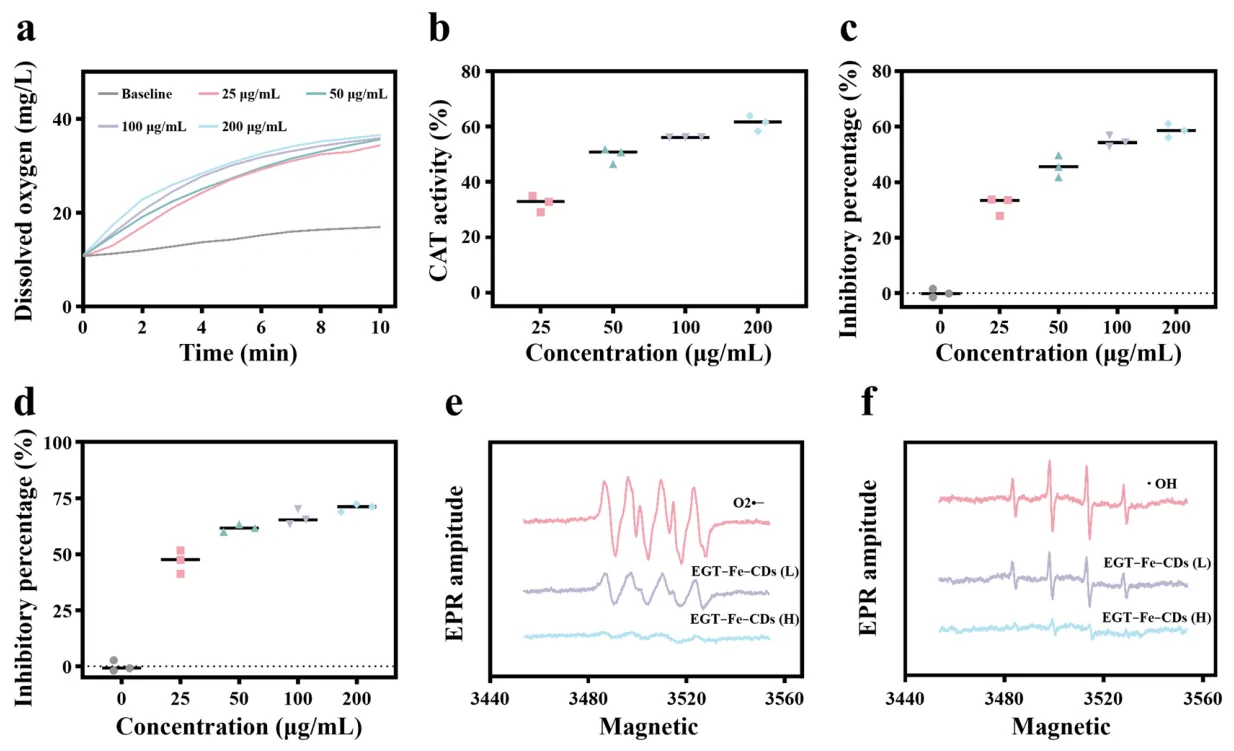
(**a**) Dissolved oxygen generation curves resulting from the decomposition of H_2_O_2_ catalysed by EGT-Fe-CDs at different concentrations (25, 50, 100, and 200 μg/mL). (**b**) CAT-like activity of EGT-Fe-CDs as a function of concentration. (**c**) Total antioxidant capacity (T-AOC) of EGT-Fe-CDs measured by the ABTS method. (**d**) Superoxide dismutase (SOD)-like activity of EGT-Fe-CDs, expressed as the inhibition rate of superoxide anion radicals (O_2_^•−^). (**e**) Electron paramagnetic resonance (EPR) measurement of the scavenging effect of EGT-Fe-CDs on superoxide anions (O_2_^•−^). (**f**) EPR measurement of the scavenging effect of EGT-Fe-CDs on hydroxyl radicals (•OH).

Low nutrient or oxygen availability can disturb redox balance in NPCs and increase ROS production. Keeping ROS in check is very important for the stability of NPCs. SOD and CAT are common antioxidant enzymes. They work step by step. First, SOD changes O_2_•^−^ into H_2_O_2_. Next, CAT turns that H_2_O_2_ into H_2_O and O_2_ [28,29]. We used a SOD activity assay kit to measure whether EGT-Fe-CDs can clean up O_2_•^−^. The results are clear. EGT-Fe-CDs break down H_2_O_2_ to make O_2_ very well. This ability depends heavily on the concentration used. We tested a range from 25 to 200 μg/mL. More dissolved oxygen appeared as the concentration went up. It hit about 40 mg/L within 10 min. The CAT-like activity followed the same pattern. It reached nearly 60% at 200 μg/mL. This number was much higher than that of the low-concentration group (Figure 2a,b). Cleaning up superoxide anions (O_2_•^−^) also relied on concentration. From 25 to 200 μg/mL, the total antioxidant capacity of the EGT-Fe-CDs grew a lot as we added more material (Figure 2c). The power to remove O_2_•^−^ went up with higher doses too (Figure 2d). Electron paramagnetic resonance (EPR) spectroscopy confirmed these facts (Figure 2e,f). The scans proved that EGT-Fe-CDs really do wipe out O_2_•^−^ and •OH. The EPR signal intensity dropped a lot in the high-concentration EGT-Fe-CDs (H) group compared to the control group. The material cleared •OH even better than O_2_•^−^. This matches the inhibition rates we saw in our other tests. All these findings mean one thing. We successfully made a new carbon-dot nanozyme (EGT-Fe-CDs) that does multiple jobs. It acts like both SOD and CAT. It also has a huge total antioxidant capacity. Because of this, EGT-Fe-CDs hold great promise for treating IVDD. They can fix the bad ROS microenvironment linked to degenerative disc disease. Additionally, the Fe-CDs group without EGT and the EGT-CDs group without Fe were also prepared. The results (Figure S1) showed that Fe ions played a major role in CAT-like activity, while both Fe ions and EGT contributed comparably to SOD-like activity. It is worth noting that EGT-Fe-CDs did not produce free radicals under the test conditions (Figure S2).

### 3.3. Effects of EGT-Fe-CDs on H_2_O_2_-Induced NPC Injury

**Figure 3:**
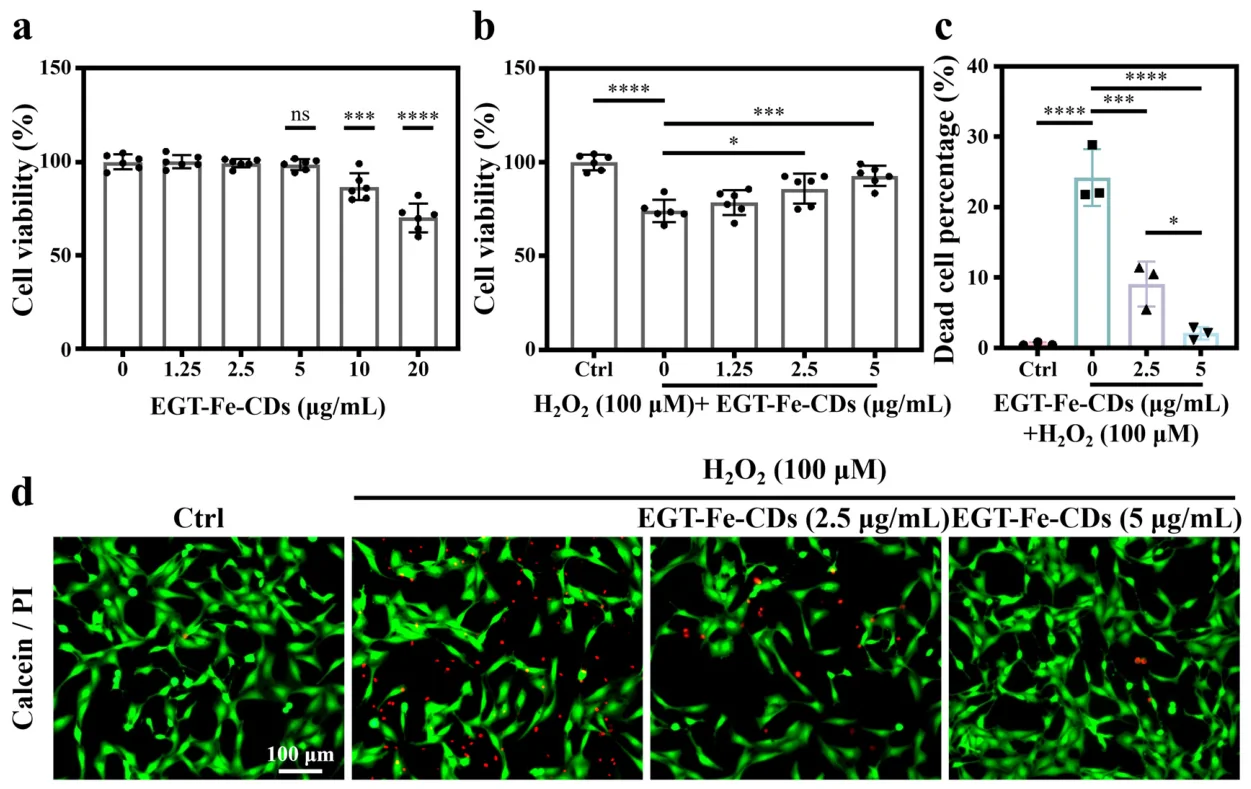
(**a**) NPC viability after treatment with different EGT-Fe-CDs concentrations, measured by CCK-8. (**b**) NPC viability after EGT-Fe-CDs treatment under H_2_O_2_ (100 μM) exposure. (**c**) Percentage of dead cells based on calcein-AM/PI staining. (**d**) Representative calcein-AM/PI images (green, live cells; red, dead cells; scale bar = 100 μm). Data are presented as mean ± standard deviation from three independent experiments (n = 3). ns indicates no significant difference; * *p* < 0.05, *** *p* < 0.001, **** *p* < 0.0001.

We first assessed EGT-Fe-CDs cytotoxicity in NPCs with a CCK-8 assay (Figure 3a). At 0–5 μg/mL, cell viability remained above 95% and did not differ significantly from the control (*p* > 0.05). At 10–20 μg/mL, viability decreased but remained above 70%, indicating low cytotoxicity [30]. After that, we built an H_2_O_2_-induced oxidative stress model to test how well EGT-Fe-CDs protect the cells (Figure 3b) [31,32]. Giving the cells 100 μM H_2_O_2_ for 24 h caused a big drop. After 100 μM H_2_O_2_ treatment for 24 h, cell viability decreased to about 75% (*p* < 0.0001). With 1.25–5 μg/mL EGT-Fe-CDs, viability increased with concentration and reached about 90% (*p* < 0.05). Calcein-AM/PI staining showed a similar pattern (Figure 3c,d). About 22% of cells were dead after H_2_O_2_ alone (*p* < 0.0001), compared with about 10% and 3% after 2.5 and 5 μg/mL EGT-Fe-CDs, respectively (*p* < 0.05). The lowest percentage of dead cells was observed at 5 μg/mL. The H_2_O_2_ treatment group in the fluorescence microscopy images (Figure 3d) was full of red PI-positive dead cells. By contrast, the EGT-Fe-CDs pretreatment groups had many more green calcein-AM-positive live cells. The red fluorescence went down a lot. At the tested concentrations, EGT-Fe-CDs showed low cytotoxicity and reduced H_2_O_2_-associated NPC injury.

### 3.4. Effects of EGT-Fe-CDs on ROS, Mitochondrial Function, and NPC Senescence

**Figure 4:**
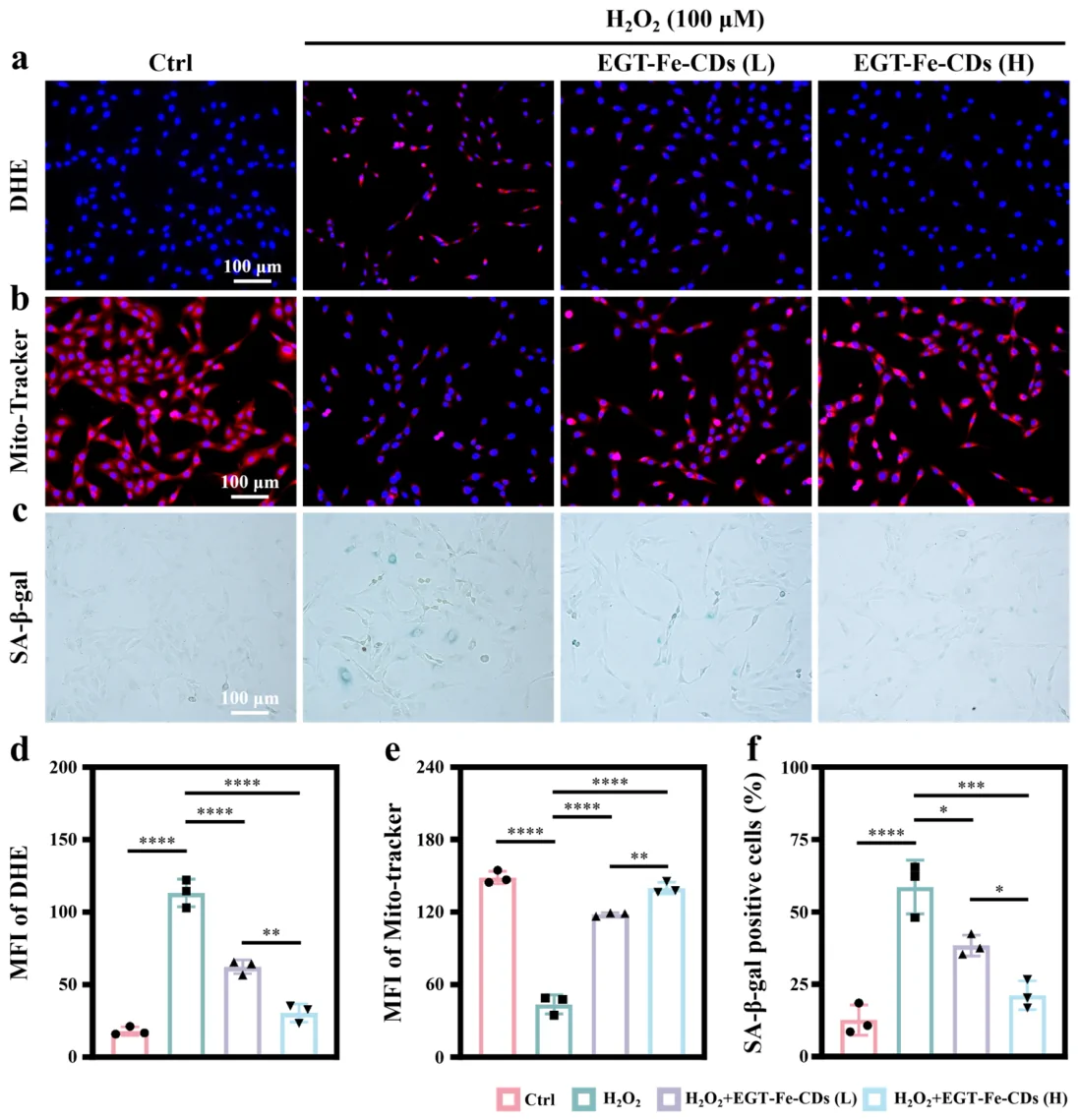
(**a**) DHE fluorescence staining to detect intracellular ROS levels in each group (red fluorescence: superoxide anion; blue fluorescence: DAPI nuclear staining; scale bar = 100 μm). (**b**) MitoTracker Red CMXRos staining to detect mitochondrial membrane potential (red fluorescence: active mitochondria; blue fluorescence: DAPI nuclear staining; scale bar = 100 μm). (**c**) SA-β-gal staining to detect cellular senescence (blue-positive cells indicate senescent cells; scale bar = 100 μm). (**d**–**f**) Quantification of DHE MFI, MitoTracker MFI, and SA-β-gal-positive cells. EGT-Fe-CDs (L, 2.5 μg/mL) and EGT-Fe-CDs (H, 5 μg/mL) denote the low- and high-concentration pretreatment groups. Data are presented as mean ± standard deviation from three independent experiments (n = 3); * *p* < 0.05, ** *p* < 0.01, *** *p* < 0.001, **** *p* < 0.0001.

We next measured intracellular ROS, mitochondrial function, and cellular senescence. DHE staining was used to examine H_2_O_2_-induced ROS in NPCs. In the presence of O_2_•^−^, DHE changes into ethylenediamine, which binds to RNA or DNA and produces red fluorescence. Higher red fluorescence therefore indicates a higher intracellular ROS level. The H_2_O_2_-treated group in Figure 4a had much stronger red fluorescence than the control group. This means H_2_O_2_ stimulation pushed ROS generation way up. After adding EGT-Fe-CDs, the red glow faded. The ROS levels clearly dropped. The high-concentration EGT-Fe-CDs [EGT-Fe-CDs(H)] group cleaned up ROS the best. Its MFI went back to almost normal (*p* < 0.01). This proves that the ROS scavenging capacity of EGT-Fe-CDs relies heavily on the dose. Too much ROS leads to oxidative stress. A broken electron transport chain in the mitochondria is the main reason for this extra ROS [33,34]. To test this, we used a mitochondrial red fluorescence probe. It specifically marks active mitochondria inside the cells. This let us measure the mitochondrial membrane potential (Figure 4b,e) [35]. Mito-Tracker Red CMXRos staining showed a big problem after H_2_O_2_ treatment. The mitochondrial membrane potential fell sharply. The MFI dropped by about 60% (*p* < 0.0001). This points to severe damage in mitochondrial function. But treating the cells first with EGT-Fe-CDs fixed this. Mitochondrial membrane potential increased after EGT-Fe-CDs pretreatment in both groups. In the high-concentration group, MFI returned to a level similar to the control (*p* < 0.01). These findings show that EGT-Fe-CDs reduced the H_2_O_2_-associated loss of mitochondrial membrane potential. Because mitochondrial redox imbalance is linked to cellular senescence and IVDD, SA-β-gal staining was then used to assess senescence (Figure 4c,f). The H_2_O_2_-treated group had a much larger percentage of senescent cells. Adding low or high doses of EGT-Fe-CDs changed the phenomenon. The number of senescent cells went down a lot in both groups. Higher doses worked better. The high-dose group had the most obvious drop (*p* < 0.001). These facts show that EGT-Fe-CDs can stop oxidative stress-induced NPC senescence. Considering all these tests, EGT-Fe-CDs offer protection on multiple levels. They clean out extra intracellular ROS fast. They keep the mitochondrial membrane potential normal. They also inhibit cellular senescence. Together, these details give solid experimental evidence explaining how they delay IVDD. 

### 3.5. Effects of EGT-Fe-CDs on Inflammatory and ECM-Related Proteins in NPCs

**Figure 5:**
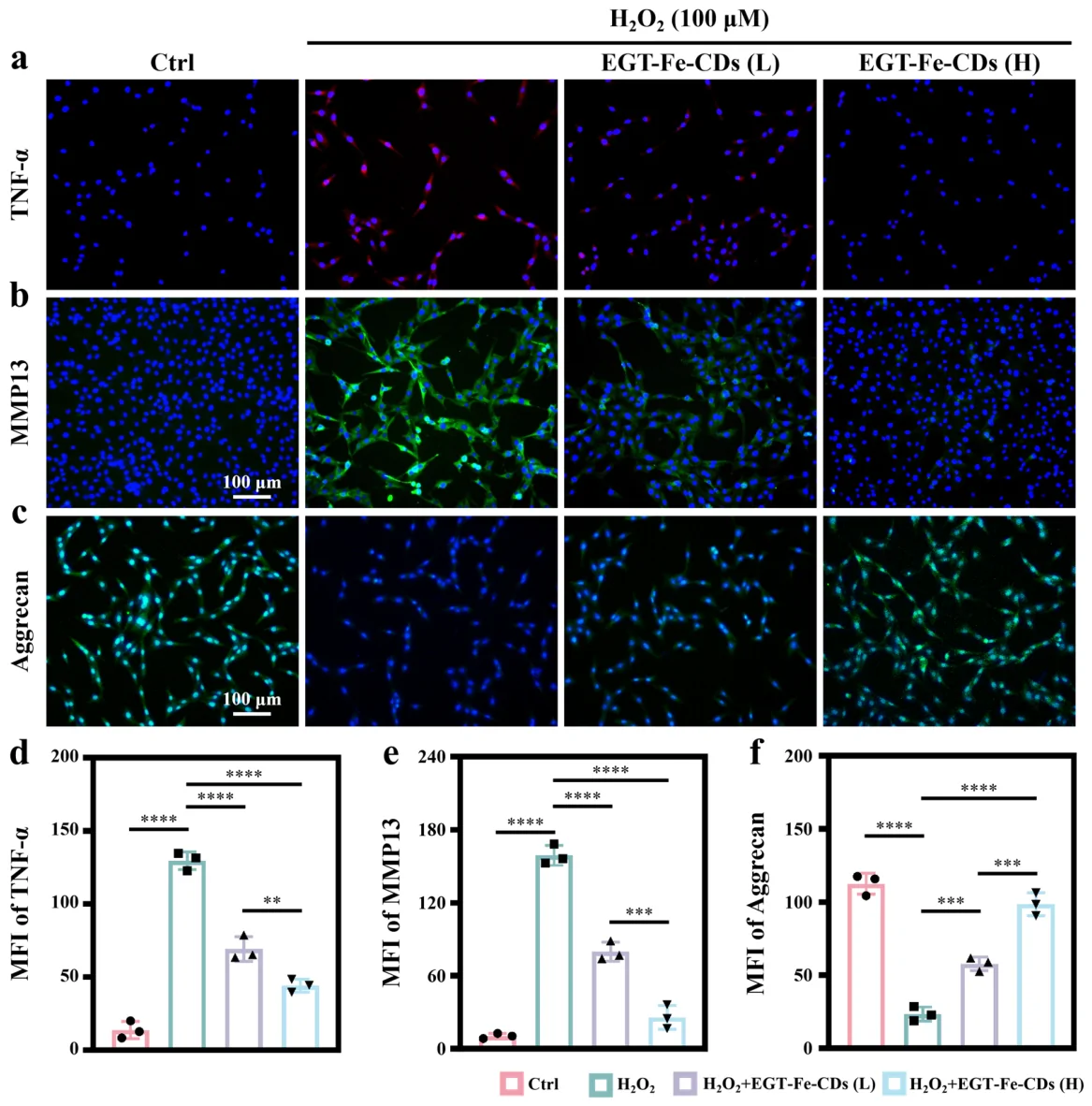
(**a**–**c**) Representative immunofluorescence images of TNF-α, MMP13, and aggrecan (red, TNF-α; green, MMP13 and aggrecan; blue, DAPI; scale bar = 100 μm). (**d**–**f**) Quantitative analysis of the mean fluorescence intensity (MFI) of TNF-α, MMP13, and aggrecan in each group. EGT-Fe-CDs (L, 2.5 μg/mL) and EGT-Fe-CDs (H, 5 μg/mL) represent the low- and high-concentration EGT-Fe-CDs pretreatment groups, respectively. Data are presented as the mean ± standard deviation from three independent experiments (n = 3); ** *p* < 0.01, *** *p* < 0.001, **** *p* < 0.0001.

ROS-related cell senescence is accompanied by secretion of inflammatory factors and proteins involved in extracellular matrix (ECM) degradation. These secreted factors are known as the senescence-associated secretory phenotype (SASP) [36]. We therefore measured selected inflammatory and matrix-related proteins after H_2_O_2_ treatment. H_2_O_2_ increased TNF-α and MMP13 expression. At the same time, anabolic protein expression decreased (Figure 5a–f). After EGT-Fe-CDs treatment, TNF-α and MMP13 expression decreased as the concentration increased. In the high-concentration group, TNF-α and MMP13 decreased by about 70% (*p* < 0.01) and 80% (*p* < 0.0001), respectively. Aggrecan increased and returned to near the control level (*p* < 0.001). Together, these changes indicate lower inflammatory and catabolic marker levels and higher anabolic marker expression in NPCs [37,38].

### 3.6. In Vivo Treatment of IVDD with EGT-Fe-CDs Through Their Antioxidant and Anti-Inflammatory Effects

**Figure 6:**
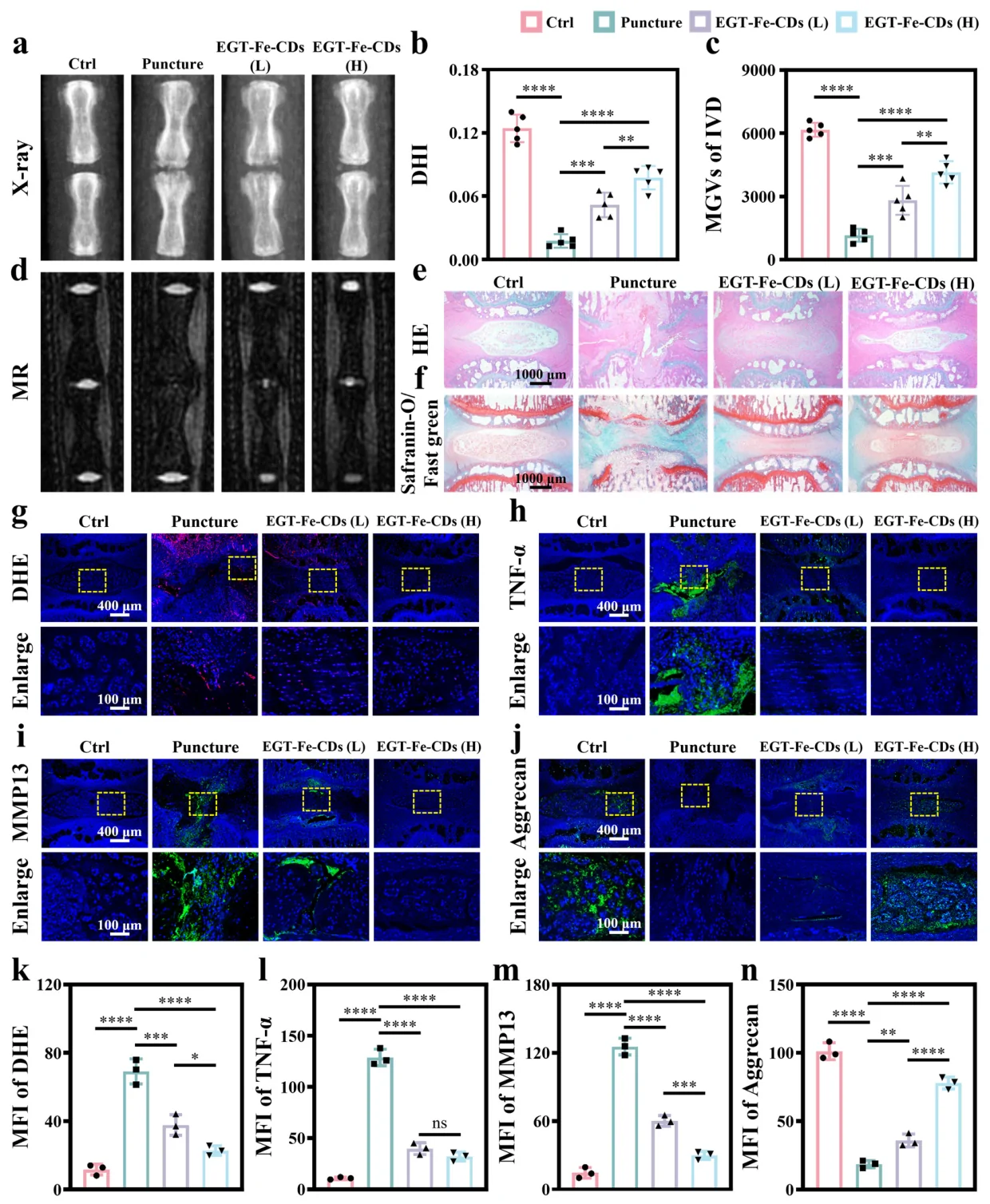
(**a**) Representative X-ray images of each group. (**b**) Quantitative analysis of the disc height index (DHI). (**c**) Analysis of mean grey values (MGVs) of the nucleus pulposus in T_2_-weighted MR images. (**d**) Representative MR images from each group. (**e**,**f**) H&E staining and Safranin O–Fast Green staining (scale bar = 1000 μm). (**g**–**j**) Immunofluorescence staining and magnified images (4× magnification) of DHE, TNF-α, MMP13, and aggrecan in each group (scale bar = 400 μm). (**k**–**n**) Quantitative analysis of the mean fluorescence intensity (MFI) of DHE, TNF-α, MMP13, and aggrecan in each group. EGT-Fe-CDs (L, 2.5 μg/mL) and EGT-Fe-CDs (H, 5 μg/mL) represent the low-dose and high-dose treatment groups, respectively. Data are presented as the mean ± standard deviation from three independent experiments (n = 3); * *p* < 0.05, ** *p* < 0.01, *** *p* < 0.001, **** *p* < 0.0001; ns indicates no statistical significance.

To evaluate if EGT-Fe-CDs actually worked inside living bodies, a rat caudal intervertebral disc needle puncture model was used to cause IVDD. Right after making the puncture, we injected EGT-Fe-CDs locally. Firstly, the biodistribution of EGT-Fe-CDs in injured disc tissue was measured through the in vivo imaging systems. The results (Figure S3) demonstrated that a distinct fluorescent signal was still detectable at the injection site three days after the administration of EGT-Fe-CDs into the injured rat intervertebral disc. Meanwhile, no obvious fluorescent signal was observed in the major organs of the rats (such as the heart, liver, spleen, lungs, and kidneys). These findings indicate that EGT-Fe-CDs can be effectively retained at the target tissue (i.e., the injured intervertebral disc) with minimal systemic distribution, thereby supporting the proposed mechanism of local therapeutic action. Four weeks later, we checked the disc damage using imaging and histology (Figure 6a–c). The X-ray results were clear. The disc height index (DHI) in the puncture group dropped by about 75% compared to the control group (*p* < 0.0001). This massive drop means the disc height was severely lost. But the EGT-Fe-CDs treatment groups looked much better. The DHI bounced back depending on the dose. The low-concentration group recovered by about 50% (*p* < 0.001). The high-concentration group did even better, returning to 70% of the normal level (*p* < 0.01). The puncture group in the T2-weighted MR images had a very weak nucleus pulposus signal. The mean grey values (MGVs) fell by almost 80% (*p* < 0.0001). This shows that the nucleus pulposus lost a lot of its water. Treatment with EGT-Fe-CDs brought the signal intensity right back up. In fact, the MGVs in the high-concentration group went up roughly threefold (*p* < 0.001). The tissue changes perfectly matched these imaging scans (Figure 6e). Under H&E staining, the control group showed a healthy, intact intervertebral disc. The line between the nucleus pulposus and the annulus fibrosus was clear. The annulus fibrosus was arranged in neat order. The puncture group was totally different. Its disc height was greatly reduced. The annulus fibrosus looked messy, and the nucleus pulposus cells and matrix suffered terrible damage. EGT-Fe-CDs fixed a lot of these problems. In the high-concentration group, the boundary between the two parts became clear again. The shape of the tissue looked almost normal. We also used Safranin O–Fast Green staining. In the puncture group, the soft chondroid structure of the nucleus pulposus was mostly gone. Bone-like tissue showed up, and the disc collapsed. The EGT-Fe-CDs treatment groups kept this chondroid structure safe. The high-concentration group had the most obvious success. Next, we did immunofluorescence staining on the intervertebral disc tissue (Figure 6f–n). DHE staining showed huge amounts of ROS in the puncture group (*p* < 0.0001). After treating the rats with EGT-Fe-CDs, the ROS levels dropped based on the dose. For the high-concentration group, ROS went down to near-normal levels (*p* < 0.05). TNF-α staining proved there was a huge inflammatory response in the puncture group (*p* < 0.0001). Giving the rats high concentrations of EGT-Fe-CDs pushed TNF-α expression down to almost normal (*p* < 0.0001). In the puncture group, MMP13 went way up, and aggrecan went way down *(p* < 0.0001). EGT-Fe-CDs treatment shifted both markers toward control levels. In the high-concentration group, MMP13 decreased by about 75% (*p* < 0.001), while aggrecan increased about threefold (*p* < 0.01). Together with the ROS and TNF-α results, these findings show that local EGT-Fe-CDs treatment was associated with less disc degeneration in the rat puncture model, lower oxidative and inflammatory markers, and a more favourable ECM marker profile. These findings support further evaluation of EGT-Fe-CDs for IVDD.

## 4. Discussion

Ageing happens because physical, social, genetic, and environmental factors build up over a long time. People usually split the reasons for ageing into two main groups. The first idea is that ageing is programmed by our genes as we grow. It happens when cells get old and when the immune and neuroendocrine systems change. The second idea blames random damage. This includes body mutations and the buildup of ROS. Truthfully, both of these processes are still not fully understood. Past studies point to several triggers. Telomere shortening, DNA damage, abnormal oncogenes, metabolic problems, and making too much ROS can all stop cells from multiplying. When cells stop growing, their shape changes. They also show higher lysosomal β-galactosidase activity and overexpress p16 and p21. Finally, they release senescence-associated secretory phenotype (SASP) factors like IL-6 and TNF-α. ROS are actually the main drivers of this ageing state. They are just byproducts of oxygen metabolism. Even so, they control cell growth, multiplication, and differentiation by changing proteins. 

Ageing NPCs are a major sign of IVDD. This ageing happens directly because too much intracellular ROS causes severe oxidative stress. Poor mitochondrial function in NPCs definitely starts and worsens IVDD. Broken mitochondria pump out abnormal amounts of reactive oxygen species. This starts a bad cycle of cellular senescence and apoptosis. Harmful factors make intervertebral disc cells produce way more ROS than they can clean up. This triggers oxidative stress. From there, inflammation starts, catabolism goes up, and the disc microenvironment falls apart. As a result, the number of healthy, viable cells drops fast. Senescent cells pile up. All of this pushes IVDD to get worse. Nanozymes are special nanomaterials that act just like real enzymes. They can copy the catalytic jobs of natural enzymes like SOD and CAT. Natural enzymes can be fragile. Nanozymes, on the other hand, have strong structural stability and flexible designs. This makes them very useful for biomedicine. 

For this work, we chose ergothioneine (EGT). It is a natural antioxidant. We used it as the carbon source to make a new carbon-dot nanozyme (EGT-Fe-CDs) through a simple hydrothermal method. EGT is a unique thiohistidine derivative that cleans up free radicals very well. However, it cannot be easily used in clinics. It has poor stability, leaves the body too fast, and suffers from low bioavailability. The EGT-Fe-CDs in this study fix these issues. During the hydrothermal reaction, they keep the active groups of EGT. The final nanostructure is loaded with surface functional groups and works like an enzyme. The assays showed the antioxidant activity of EGT-Fe-CDs. Their particle size was about 3–5 nm, and hydroxyl, amino, and thiol groups were present on the surface. Iron ion complexes were also detected and may contribute to enzyme-like catalysis. In cell experiments, EGT-Fe-CDs reduced ROS, maintained mitochondrial membrane potential, and lowered inflammatory factors related to the senescence-associated secretory phenotype. We still need to figure out the exact molecular rules behind this. Finding those exact answers will guide the smart design and targeted changes for EGT-Fe-CDs in the future.

## 5. Conclusions

Making EGT-Fe-CDs through hydrothermal synthesis is simple and cheap. Compared to natural enzymes or standard carbon dots, these EGT-Fe-CDs have much stronger multienzyme activity. EGT-Fe-CDs showed both SOD-like and CAT-like activities. The material retained active groups from the EGT precursor and included metal ion doping, and it showed efficient ROS scavenging. In NPC experiments, EGT-Fe-CDs had low cytotoxicity and reduced H_2_O_2_-associated injury, ROS accumulation, loss of mitochondrial membrane potential, and cellular senescence. Matrix synthesis increased, while catabolic enzymes and inflammatory factors decreased. In the rat tail puncture model, EGT-Fe-CDs treatment was associated with better DHI, MRI signal intensity, and Pfirrmann grade. Histology showed better preservation of the nucleus pulposus and annulus fibrosus, and the in vivo ROS and inflammatory protein findings were consistent with the cell experiments. The material was safe and was cleared rapidly in vivo. Overall, these results support further study of EGT-Fe-CDs as a nanozyme approach for IVDD. Further studies may examine whether similar effects occur in other oxidative stress-related diseases.

## Data Availability

The original contributions presented in this study are included in the article/Supplementary Material. Further inquiries can be directed to the corresponding authors.

## Supplementary Materials

The following supporting information can be downloaded at: https://www.mdpi.com/article/10.3390/antiox15091082/s1, Figure S1. Dissolved oxygen generation curves resulting from the decomposition of H_2_O_2_ catalyzed by (a) EGT-Fe-CDs, (b) EGT-CDs, (c) Fe-CDs at different concentrations (25, 50, 100, and 200 μg/mL). Superoxide dismutase (SOD)-like activity of (d) EGT-Fe-CDs, (e) EGT-CDs, (f) Fe-CDs.; Figure S2. The peroxidase-like (POD-like) activity of EGT-Fe-CDs. Figure S3. In vivo imaging results of EGT-Fe-CDs.
