# Supplementary material for: Ergothioneine-Derived Carbon Dot Nanozymes Alleviate the Oxidative Stress Microenvironment to Delay Intervertebral Disc Degeneration
Source: Antioxidants (Basel). 2026 Aug 28;15(9):1082. doi: 10.3390/antiox15091082 (PMC13603896; doi:10.3390/antiox15091082)
Supplement: Supplementary file 1 [file antioxidants-15-01082-s001.zip › antioxidants-4438122-supplementary.pdf]

Supporting Information

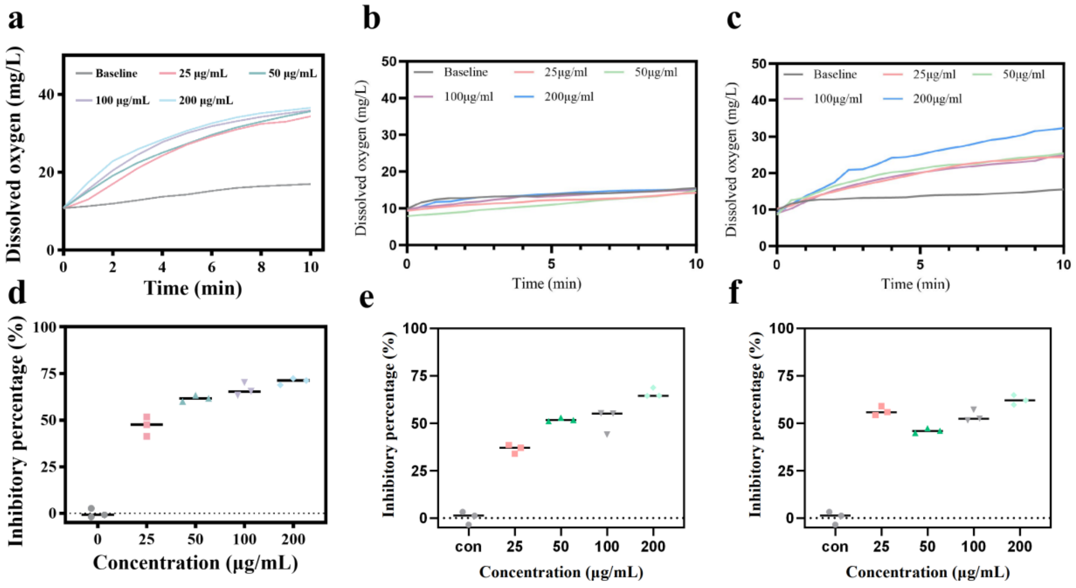

Figure S1. Dissolved oxygen generation curves resulting from the decomposition of H_2O_2 catalyzed by (a) EGT-Fe-CDs, (b) EGT-CDs, (c) Fe-CDs at different concentrations (25, 50, 100, and 200 μg/mL). Superoxide dismutase (SOD)-like activity of (d) EGT-Fe-CDs, (e) EGT-CDs, (f) Fe-CDs.

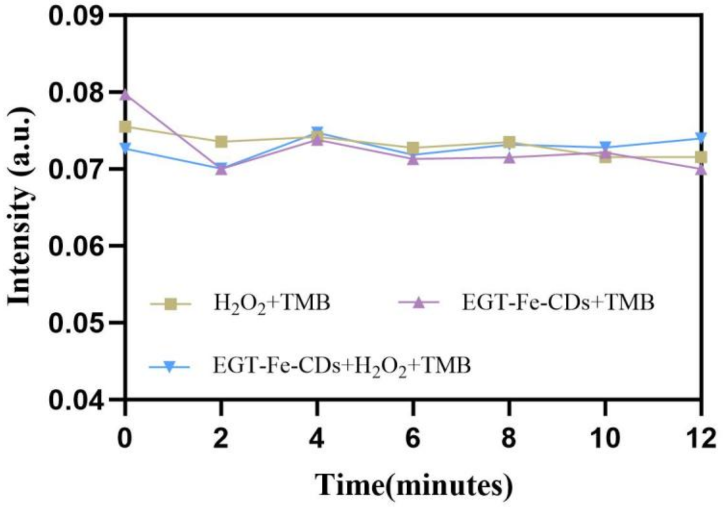

Figure S2. The peroxidase-like (POD-like) activity of EGT-Fe-CDs.

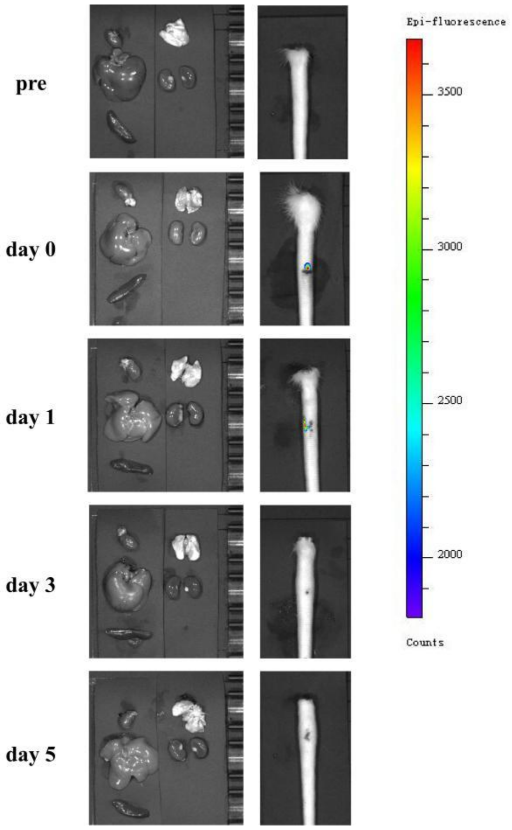

Figure S3. In vivo imaging results of EGT-Fe-CDs.
